# Effect of Seed Treatment by Cold Plasma on the Resistance of Tomato to *Ralstonia solanacearum* (Bacterial Wilt)

**DOI:** 10.1371/journal.pone.0097753

**Published:** 2014-05-19

**Authors:** Jiafeng Jiang, Yufang Lu, Jiangang Li, Ling Li, Xin He, Hanliang Shao, Yuanhua Dong

**Affiliations:** 1 Key Laboratory of Soil Environment and Pollution Remediation, Institute of Soil Science, Chinese Academy of Sciences, Nanjing, P. R. China; 2 State Key Laboratory of Soil and Sustainable Agriculture, Institute of Soil Science, Chinese Academy of Sciences, Nanjing, P. R. China; 3 University of Chinese Academy of Sciences, Beijing, P. R. China; 4 Changzhou ZhongKe ChangTai Plasma Technology Co., Ltd., Changzhou, P. R. China; University Paul Sabatier, France

## Abstract

This study investigated the effect of cold plasma seed treatment on tomato bacterial wilt, caused by *Ralstonia solanacearum* (*R. solanacearum*), and the regulation of resistance mechanisms. The effect of cold plasma of 80W on seed germination, plant growth, nutrient uptake, disease severity, hydrogen peroxide (H_2_O_2_) concentration and activities of peroxidase (POD; EC 1.11.1.7), polyphenol oxidase (PPO; EC 1.10.3.2) and phenylalanine ammonia lyase (PAL; EC 4.3.1.5) were examined in tomato plants. Plasma treatment increased tomato resistance to *R. solanacearum* with an efficacy of 25.0%. Plasma treatment significantly increased both germination and plant growth in comparison with the control treatment, and plasma-treated plants absorbed more calcium and boron than the controls. In addition, H_2_O_2_ levels in treated plants rose faster and reached a higher peak, at 2.579 µM gFW^−1^, 140% greater than that of the control. Activities of POD (421.3 U gFW^−1^), PPO (508.8 U gFW^−1^) and PAL (707.3 U gFW^−1^) were also greater in the treated plants than in the controls (103.0 U gFW^−1^, 166.0 U gFW^−1^ and 309.4 U gFW^−1^, respectively). These results suggest that plasma treatment affects the regulation of plant growth, H_2_O_2_ concentration, and POD, PPO and PAL activity in tomato, resulting in an improved resistance to *R. solanacearum*. Consequently, cold plasma seed treatment has the potential to control tomato bacterial wilt caused by *R. solanacearum*.

## Introduction

Tomato (*Solanum lycopersicum* L.) is one of the most important economic crops in the world. Each year, bacterial wilt, caused by *Ralstonia solanacearum,* results in huge reductions in tomato yield [1]. The pathogen enters plant roots through natural openings or wounds and invades the xylem vessels. It then proliferates and spreads throughout the vascular system, secreting a large amount of extra-cellular polysaccharides that prevent water transportation, leading, ultimately, to plant death. Various strategies have been developed for the control of bacterial wilt, including the development of resistant varieties [2], use of chemical fumigants [3], soil drainage [4], and specific tillage practices [5]. However, these current methods each have their own limitations, due to a variety of reasons.

Cold plasma seed treatment is a modern eco-agricultural technology that has been suggested to stimulate plant growth. It is based on non-ionizing low-level radiation (so called cold plasma), which can activate the vitality of seeds but without causing gene mutations, and is quite different from space breeding or mutation breeding by particle beam. Zivkovic *et al.* [6] demonstrated that cold air plasma pretreatment significantly improved the germination of *Paulownia tomentosa*. Cold plasma seed treatment has also been reported to improve the growth and yield of wheat [7]. Concomitant with the promotion of growth, plant resistance to biotic and abiotic stresses was also increased. Since cold plasma treatment can improve the growth of the plant, it may have an additional impact on its disease resistance.

Early work demonstrated that seed sterilization could be achieved through cold plasma treatment, which can kill bacterial cells or the spores of several *Bacillus* spp. [8]–[10]. Two major mechanisms are involved in the inactivation of microorganisms by plasma treatment: direct destruction by UV irradiation of microorganisms [11], and erosion of the microorganisms by oxygen atoms or radicals emanating from the plasma itself [12]. Previous studies have also shown that plasma treatment could increase dehydrogenase activity and peroxidase activities of tomato seedlings [13]. However, research into the effect of plasma treatment on plant disease resistance is rare.

Hence, the question still remains as to whether plasma treatment could affect the plant’s physiological response to disease, leading to disease alleviation. The aim of the present study was to investigate the effect of cold plasma treatment on tomato growth, and also, the resistance of tomato to bacterial wilt. This involved: (i) investigation into the effect of cold plasma treatment on plant growth, severity of disease development, hydrogen peroxide (H_2_O_2_) concentration and resistance-related enzyme activity in tomato; and (ii) identification of the regulatory mechanisms underlying tomato bacterial wilt resistance that are altered by cold plasma treatment.

## Materials and Methods

### Ethics Statement

No specific permits were required for the described field studies. The location studied is not privately-owned or protected in any way, and the field studies did not involve endangered or protected species.

### Experimental Apparatus

The apparatus for seed plasma treatment was described in our previous research work [7]. The experiments were performed in the commercial computer-controlled plasma treatment apparatus HD-2N. The system was consisted with vacuum system, which would vacuum the processing system by rotary pump, and plasma generator. Capacitively coupled plasma (CCP) was generated by RF discharge. Seeds were exposed to inductive helium plasma discharge under the following parameters: the plasma frequency was 13.56 MHz, the power was 80W, the pressure was 150 Pa and the volume of the discharge chamber was 1200 mm * 180 mm * 20 mm. The time span of irradiation was 15 s and the temperature of the discharge, measured by a thermistor, was about 25°C.

### Plasma Treatment

Tomato seeds were overspread at the bottom of glass petri dish (150 mm in diameter), and they were kept not touching each other, then the petri dish was placed into the apparatus. Seeds were exposed to inductive plasma treatment with RF discharge. Meanwhile, without plasma treatment, control seeds were also submitted to the same vacuum and helium flux as the treated seeds. Experiments of plant were carried out approximately one day after seeds were treated with cold plasma.

### Seed Germination Assay

Seeds of *S. lycopersicum* L. cv. Shanghai 906, susceptible to bacterial wilt, were subjected to cold plasma treatment. The seed germination assay was based on the standards for agricultural seed testing, Germination test (GB/T 3543.4-1995) of national standard of the People’s Republic of China. 100 seeds, placed in each culture dish evenly, were cultured in 25°C light incubator for 7 days, and the number of seed germination was observed and recorded every day. The standard to determine seed germination was that the germ length reached half of the seed length. The experiment setup 4 repeats.

List of investigated characteristics (with all used indices):

N_TS_: Number of total seeds per dish.

N_D3_: Number of germinated seeds per dish in 3 day

N_D7_: Number of germinated seeds per dish in 7 day

GP: Germination potential

GR: Germination rate*i* Disease index

*i_max_*: Highest disease index

N_TP_: Total number of plants investigated

N_P*i*_: Number of plants with disease index of i

DS: Disease Severity
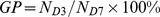





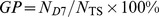



### Greenhouse Experiment

The yellow-brown earth (Argosols) used in the greenhouse experiment was collected from a field that suffered from severe wilt diseases, with continuous vegetable cultivation of over 5 years in Qixia District (32°08′N, 118°54′E), Nanjing, Jiangsu Province, China. Cold plasma treated tomato seeds were sown and seedlings were grown in a mixture of vermiculite and perlite (1∶1 v/v). Seedlings were fertilized twice weekly with Hoagland’s Solution commencing 1 week after sowing. After 30 days, seedlings were transplanted to individual pots, 12 cm in height and 12 cm in diameter, filled with 1 kg of soil. Each pot was fertilized with 0.57 g urea, 0.11 g KCl and 0.09 g Ca(H_2_PO_4_)_2_ before transplanting. There were three replicates with 36 seedlings per treatment. Plants were grown in a greenhouse maintained between 15°C to 25°C under natural light, and watered regularly to maintain soil water content as 70% of field maximum moisture capacity.

### Plant Growth

Plant height, leaf thickness, stem diameter, leaf area, dry weight and chlorophyll content were measured 30 days after transplantation into individual pots. Chlorophyll content was assayed as described by Porra *et al.* [14]. Briefly, chlorophyll was extracted from 50 mg of tomato leaf tissue in 1 mL of 80% (v/v) acetone containing 2.5 mM sodium phosphate buffer (pH 7.8) on ice. After centrifugation at 4°C for 20 min at 16,000 × *g*, absorbance at 663.6, 646.6 and 750 nm was measured with a spectrophotometer (DU800, Beckman, USA).

### Plant Nutrients Analysis

The non-inoculated tomato plants were harvested, washed with distilled water and dried at 70°C for 2 h, and 105°C for 48 h. Amounts of 100 mg of each dried sample were digested with a nitric acid-perchloric acid mixture (HNO_3_-HClO_4_). Nitrogen was analyzed using the total Kjeldahl nitrogen method. The filtrate was analyzed for phosphorus (P), zinc (Zn), manganese (Mn), aluminum (Al), iron (Fe), nitrogen (N), potassium (K), magnesium (Mg), boron (B) and calcium (Ca) using an atomic absorption spectrometer (model AA-670, Shimadzu Co., Kyoto, Japan).

### Pathogen Inoculation and Disease Assessment

Bacterial strain and growth condition was described as Li *et al.* [15]. After incubation, the suspension population was counted in a bacterial counting chamber and adjusted to 10^8^ cells mL^−1^. The bacterial suspension (10 mL) was poured onto the soil near the roots of the tomato seedlings, at 30 days of growth. Disease development was scored every other day for 20 days after inoculation by visual observation, with a rating scale of 0 to 4, in which 0 =  no symptoms observed; 1 =  light mottling and a few thin yellow veins; 2 =  mottling and vein clearing unevenly distributed on the leaf; 3 =  mottling, leaf distortion and stunting; and 4 =  severe mottling, leaf curling and stunting [16]. Disease severity was calculated as follows:
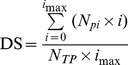



### H_2_O_2_ Concentration Measurement

Leaf samples were collected 0 (just before inoculation), 6, 12, 24, 48, 72 and 96 h after inoculation, with four replicates for each treatment. The concentration of H_2_O_2_ in the tissue was determined following the method described by Kar and Choudhuri [17]. H_2_O_2_ was isolated from 1 g leaf tissue in ice-cold acetone. 5% (w/v) of titanium sulfate and ammonium hydroxide solution was added to precipitate the peroxide-titanium complex. The precipitate was collected by centrifugation at 3,000 rpm for 10 min, and dissolved in 2 M sulfuric acid. The absorbance of the solution was determined by a spectrophotometer (DU800, Beckman, USA). The H_2_O_2_ concentration was calculated from a standard curve prepared in the same manner.

### Enzyme Assay

Leaf samples were collected 0 (just before inoculation), 6, 12, 24, 48, 72 and 96 h after inoculation (four replicates for each treatment). Samples were immediately flash-frozen and stored in liquid nitrogen prior to analysis. Peroxidase (POD; EC 1.11.1.7) and polyphenol oxidase (PPO; EC 1.10.3.2) were extracted as follows: 1.0 g of leaf tissue was homogenized in 5 mL of phosphoric acid extracting buffer (0.05 M phosphate, pH 7.0) in an ice bath. The homogenates were filtered through four layers of cheese cloth and collected after centrifugation at 12,000 rpm for 20 min at 4°C, and the supernatants used for the enzyme activity assays. Phenylalanine ammonia lyase (PAL; EC 4.3.1.5) was extracted in a similar manner, except that the buffer consisted of 0.05 M boric acid, 5 mM mercaptoethanol, 1 mM EDTA and 0.05 g of polyvinylpyrrolidone. The activity of the enzymes was assayed as described by Li *et al.* [18].

### Statistical Analysis

Data were subjected to analysis of independent-samples T test, and analyses were performed using the Statistics software package SPSS 18.0.

## Results

### Seed Germination and Plant Growth

The germination of tomato seeds was influenced by cold plasma treatment (Table 1). Germination potential and germination rate of the controls were 67% and 80% respectively, whereas cold plasma treatment increased the germination potential of tomato seeds by 8%, and significantly increased the germination rate by 11%, compared to the control.

**Table 1:** Effect of cold plasma treatment on tomato seed germination.

Treatment	Germination potential (%)	Germination rate (%)
Control	67±4a	80±5a
Plasma treatment	75±4a	91±3b

The data were expressed as the mean ± standard deviation (SD, n = 4). Different italicized letters within a column indicate significant differences as determined by T test (*P*<0.05).

The plant height, leaf thickness, stem diameter and dry weight of plants having undergone cold plasma treatment were 54.28 cm, 0.345 mm, 7.37 mm and 3.89 g, respectively, which were significantly higher than the controls by 10.8%, 10.7%, 17.2% and 9.3% respectively (Table 2). However, the chlorophyll content and leaf area of plasma-treated plants were lower than the controls.

**Table 2:** Effect of cold plasma treatment on the growth of tomato.

	Plant height (cm)	Leaf thickness (0.01 mm)	Stem diameter (mm)	Chlorophyll content (%)	Leaf area (cm^2^)	Dry weight (g)
Control	49.00±2.23a	31.17±2.23a	6.29±0.20a	19.60±0.70a	635.34±65.98a	3.56±0.09a
Plasma treatment	54.28±1.96b	34.50±2.88b	7.37±0.29b	17.97±0.31b	614.30±50.33a	3.89±0.15b

The data were expressed as the mean ± standard deviation (n = 4). Different italicized letters within a column indicate significant differences as determined by T test (*P*<0.05).

### Nutrient Uptake

Tissue P, Zn, Mn, Al and Fe of plasma-treated plants were higher than in the controls, whilst the N, K and Mg content were lower (Table 3). However, these observed differences were not statistically significant. The Ca and B content of plasma-treated plants were 23.13 g/kg and 95.50 mg/kg, respectively, significantly greater than in the control by 7.73% and 11.53%, respectively.

**Table 3:** Nutrient content of tomato plants.

	N (g/kg)	P (g/kg)	K (g/kg)	Ca (g/kg)	Mg (g/kg)	B (mg/kg)	Zn (mg/kg)	Mn (mg/kg)	Al (mg/kg)	Fe (g/kg)
Control	19.59±0.43a	3.82±0.29a	15.38±0.53a	21.47±0.23a	4.29±0.24a	85.62±3.41a	69.88±9.91a	93.18±8.87a	721.12±37.97a	1.05±0.14a
Plasma treatment	18.92±0.53a	4.07±0.23a	14.90±0.89a	23.13±0.70b	4.25±0.02a	95.50±2.02b	73.3±8.85a	106.6±10.42a	789.72±84.03a	1.11±0.11a

The data were expressed as the mean ± standard deviation (n = 4). Different italicized letters within a column indicate significant differences as determined by T test (*P*<0.05).

### Disease Severity

**Figure 1:**
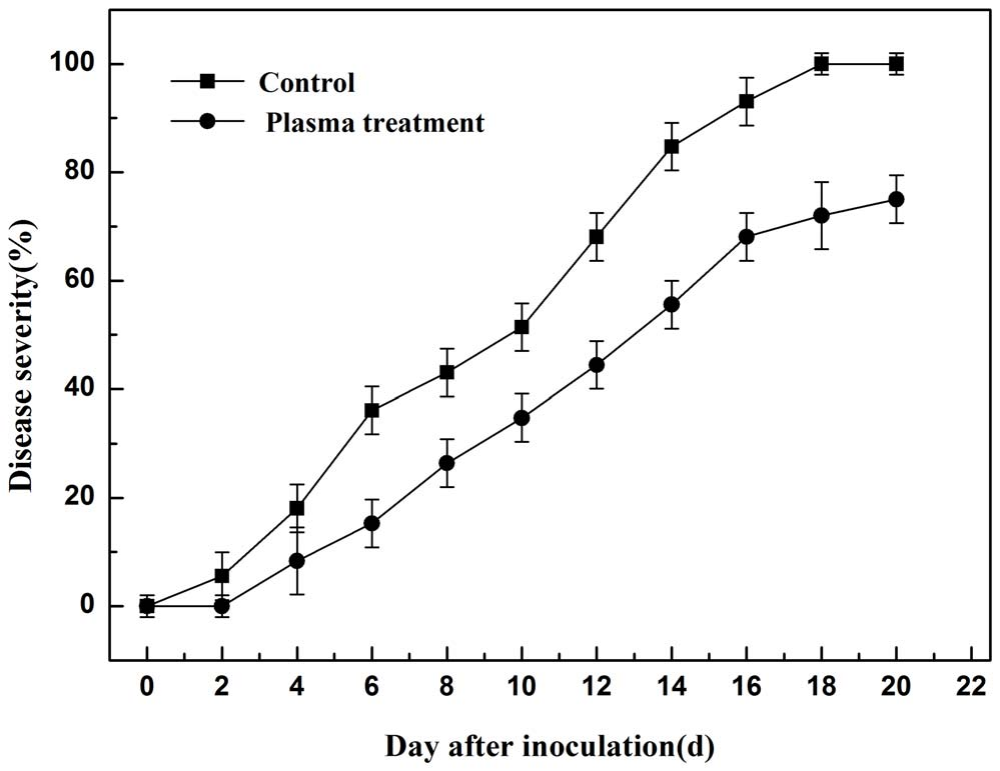
Disease development in tomato after inoculation with *R. solanacearum*.

Dynamic differences in disease severity were observed between plants having undergone cold plasma treatment and the controls, following inoculation with *R. solanacearum* (Fig. 1). Control plants began to wilt 2 days after inoculation and the disease progressed rapidly, with all plants dead 18 days after inoculation. In contrast, plasma-treated plants started to wilt 4 days after inoculation, 2 days later than the controls, and the disease progressed more slowly such that the disease severity was only 75.0% 20 days after inoculation, 25.0% less than in the controls.

### H_2_O_2_ Concentration in Tomato Tissue

**Figure 2:**
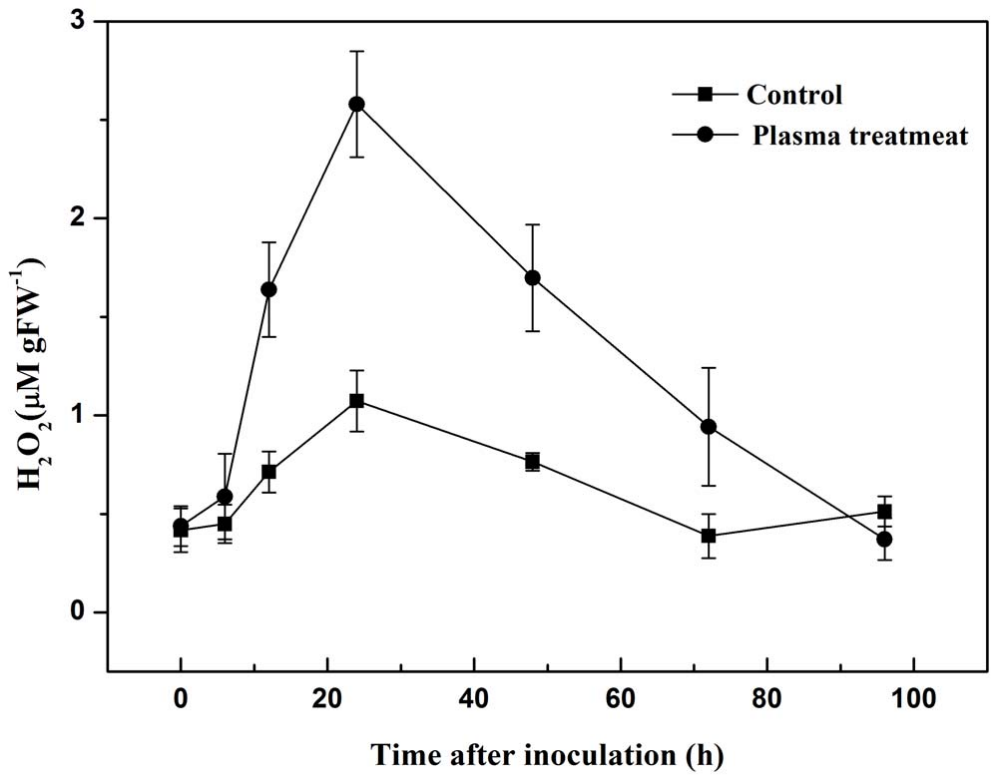
Changes in H_2_O_2_ concentration in tomato leaves from 0 to 96 h after inoculation with *R. solanacearum*.

Prior to inoculation with *R. solanacearum* the H_2_O_2_ concentration in tomato leaves was low, and was similar in both plasma-treated and control plants. However, it increased rapidly following inoculation and rose to its highest level in the first 24 h after inoculation (Fig. 2). The H_2_O_2_ concentration in leaves from plasma-treated and control plants were 2.579 and 1.073 µM gFW^−1^, respectively, which were 488% and 157%, respectively, greater than that before inoculation. From 0 to 24 h after inoculation, H_2_O_2_ concentration in plant leaves from the plasma treatment group increased faster, reaching 2.579 µM gFW^−1^, 140% greater than the control. From 24 h to 96 h after inoculation, H_2_O_2_ concentration declined slowly to the original level.

### Resistance-related Enzymes

**Figure 3:**
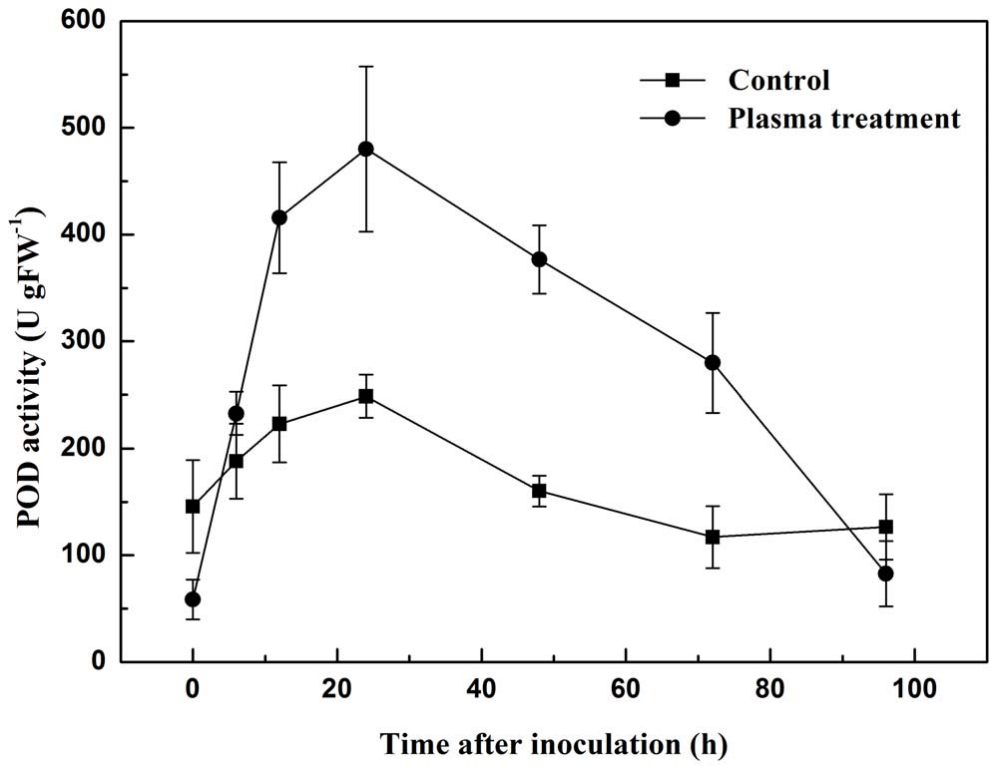
Changes in POD activity in tomato leaves 0 to 96 h after inoculation with *R. solanacearum*.

**Figure 4:**
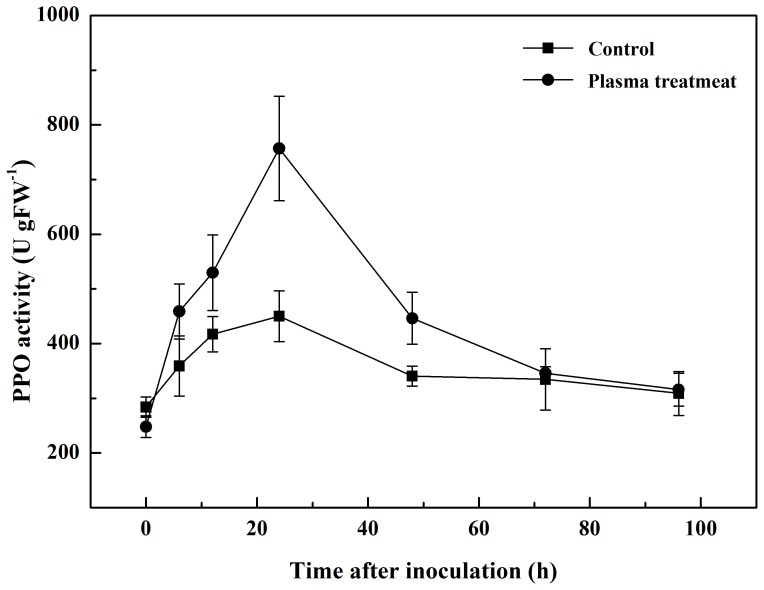
Changes in PPO activity in tomato leaves 0 to 96 h after inoculation with *R. solanacearum*.

**Figure 5:**
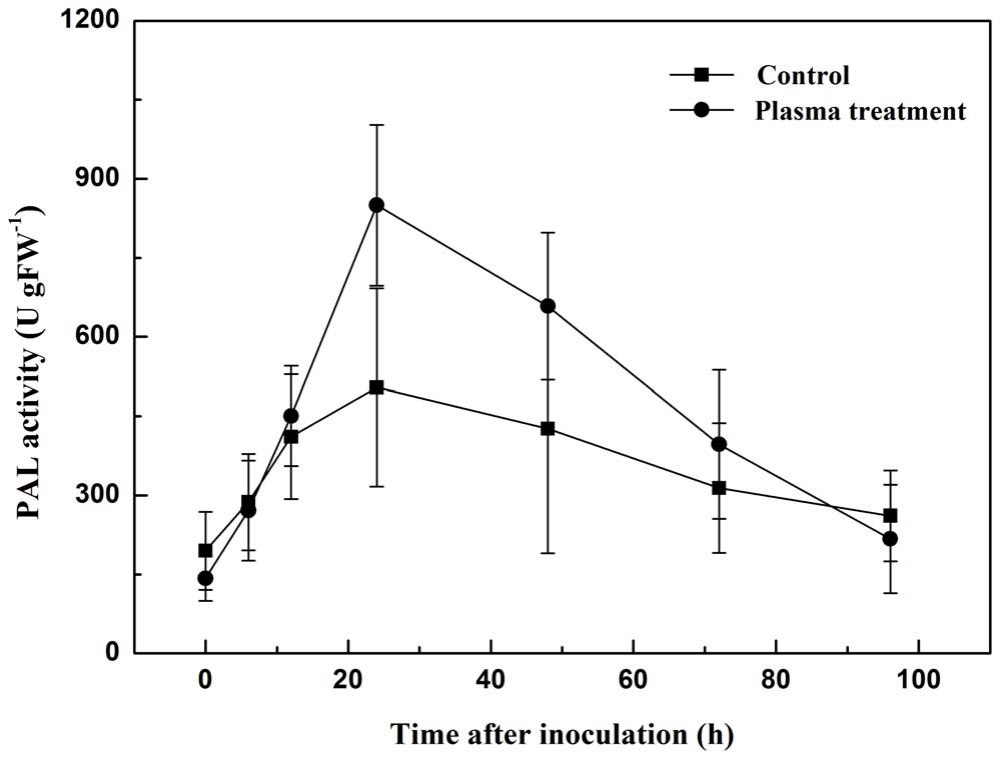
Changes in PAL activity in tomato leaves 0 to 96 h after inoculation with *R. solanacearum*.

The activities of POD, PPO and PAL were low before inoculation with the pathogen and were similar in both plasma-treated and control groups (Figs. 3, 4, 5). However, they increased to their highest point in the first 24 h after inoculation, and declined slowly to the original level from 24 h to 96 h after inoculation. At its highest level, POD activity increased to 480.0 U gFW^−1^ in the plasma treatment, 92.9% greater than that of the control, with an increment of 421.3 U gFW^−1^, significantly higher than it was 103.0 U gFW^−1^ in control. PPO activity in the control group was 449.7 U gFW^−1^, 40.6% lower than that of the plasma treatment group. And the increment was 166.0 U gFW^−1^, significantly lower than it was 508.8 U gFW^−1^ in control. In the plasma treatment, PAL activity has raised for 707.3 U gFW^−1^, 128.6% greater than that of the control. The maximal level of PAL activity was 849.8 U gFW^−1^, significantly higher than that of the control (504.3 U gFW^−1^).

## Discussion

Fast acting, economically viable and environmentally friendly methods are needed to control plant disease during agricultural activities. Currently, cold plasma stimulation plays an important role in crop optimization, through activation of endogenous substances in seeds, rejuvenation, promotion of plant growth and maximization of yield. However, little information is available on the effect of cold plasma on plant disease control. In the present study, we investigated the effect of a cold plasma pretreatment on the growth, nutrient uptake, bacterial wilt severity and physiological responses involving H_2_O_2_, POD, PPO and PAL activity in tomato. In addition, we attempted to elucidate both the effect, and mechanism of action, of cold plasma on the control of tomato bacterial wilt.

Firstly, we found that cold plasma could reduce the severity of tomato bacterial wilt, with a control efficacy of 25%. Our results showed that cold plasma could significantly improve both the germination and growth of tomato. In agreement with these results, previous studies have also demonstrated that plasma treatment could increase the germination of seeds [19], [20] and the growth of wheat and oats [21]. Consequently, it is known that the growing status of plants affects their disease resistance by altering their state of readiness to fight pathogenic attack. Our results indicate that the reduction in disease severity observed in the tomato plants pre-treated with cold plasma was partly due to their improved healthy growth.

Nutrients are essential for plant growth and are important factors in disease control [22]. Our results show that cold plasma treatment could affect the plant’s nutrient uptake. Various studies have demonstrated the mitigating effect of calcium [23], [24] and boron [25] on disease management. Since increased Ca [26], [27] and B [28] concentrations in plant tissue are associated with reduced disease symptoms, it seems likely that the observed increases in Ca and B concentrations in plants stimulated by the cold plasma treatment was responsible for alleviating the bacterial wilt of tomato. It has also been reported that disease reduction is related to the increased Ca [29] and B [30] uptake by plants.

As an important reactive oxygen species (ROS), H_2_O_2_ is generated in the early events following pathogen infection [31], and plays a key role in plant disease resistance [32]. In the present study, we observed a burst of H_2_O_2_ generation after inoculation with *R. solanacearum* in both the plasma treatment and control groups, indicating an active role for H_2_O_2_ in the tomato defense system. Plasma treatment significantly increased the accumulation of H_2_O_2_ in tomato, indicating that the reduction of disease severity was likely due to the increased H_2_O_2_ concentration.

POD is involved in the detoxification of ROS [33], and is an important antioxidant in plant cells. PPO catalyzes the oxygen-dependent oxidation of phenols to quinones, and PAL is involved in plant salicylic-acid-mediated defense against pathogens [34]. Both PPO and PAL participate in systemic acquired resistance [35]. In this study, we observed a progressive increase in POD, PPO and PAL activities in the plasma treatment and control groups during the first 24 h after inoculation, indicating that POD, PPO and PAL may play an active role in the tomato defense system. At the same time, plasma treatment significantly increased the activities of POD, PPO and PAL in plant tissue compared with the control. Henselova *et al.* [36] reported that plasma treatment could significantly increase the activities of antioxidant enzymes such as catalase (CAT) and superoxide dismutase (SOD). It has also been reported that dehydrogenase and POD activities in plants increase when treated with plasma [13]. Our data indicates that the observed reduction in plant disease symptoms was a direct result of the increased activities of POD, PPO and PAL, achieved through cold plasma treatment.

## Conclusion

In this study, we have presented a first glance into the effect and physiological regulatory mechanism underlying the role of cold plasma treatment in tomato bacterial wilt resistance. Cold plasma treatment significantly improved the growth of tomato, increased Ca and B concentrations in tomato tissues, and stimulated the production of H_2_O_2_, and the activity of POD, PPO, PAL, leading to a reduction in the severity of tomato wilt. The results presented here show future prospects for the application of cold plasma seed treatment on plant disease control. And further researches on the timeliness of plasma treatment effect, plasma exposure time and other types of discharges are needed to fully understand the specific role of cold plasma treatment on plant disease control.
